# Effect of Dilution on the Crystallization Kinetics of Neodymium-Based Rare Earth Polybutadiene Rubber

**DOI:** 10.3390/polym16010035

**Published:** 2023-12-21

**Authors:** Xiaohu Zhang, Xiaofan Li, Wenbin Zhu, Xinzheng Xie, Huan Ji, Jifu Bi

**Affiliations:** 1 Huangpu Institute of Materials, Centers for Aircraft Science, Guangzhou 510700, China; lixiaofan@ciac.ac.cn (X.L.); zhuwenbin@ciac.ac.cn (W.Z.); xiexinzheng@ciac.ac.cn (X.X.); jihuan@ciac.ac.cn (H.J.); 2 Changchun Institute of Applied Chemistry, Chinese Academy of Sciences, Changchun 130102, China

**Keywords:** neodymium-based polybutadiene rubber, dilution effect, crystallization kinetics, crystallization activation energy

## Abstract

The crystallization behavior of neodymium-based rare earth polybutadiene rubber (Nd-BR) is studied in the presence of small-molecule treated distillate aromatic extract (TDAE) and high-molecular-weight polybutadiene–isoprene copolymer rubber (BIR). Pronounced inhibitory effects on the crystallization of Nd-BR are exhibited by both materials, as evidenced by reductions in the crystallization temperature (*T*_c_), melting point (*T*_m_), and corresponding enthalpy change. It is found that, at equal concentrations, a greater influence on the crystallization rate is exerted by TDAE oils, whereas nucleation inhibition is more potently affected by BIR. Incomplete crystallization during cooling is exhibited by Nd-BR when the TDAE oil concentration reaches 40 parts per hundreds of rubber (PHR) (31 wt.%), or BIR achieves a 60 wt.% concentration; subsequently, a noticeable cold crystallization phenomenon is observed upon heating. Insights into the isothermal crystallization kinetics are offered by the data, which reveal that the Avrami index *n* value for Nd-BR predominantly ranges between 2.5 and 3.0. A decrease in the *n* value is induced by a small amount of TDAE oil, while a noticeable decline in the *n* value is observed only when the BIR concentration is 60 wt.%. A correlation between the crystallization activation energy, the concentration of TDAE oil and BIR, and the crystallization temperature is established; a negative activation energy is recorded, and a decrease in the crystallization rate is noted when both concentrations are low and the crystallization temperature exceeds −50 °C. In contrast, positive activation energy and an increase in the crystallization rate are observed when the BIR concentration reaches 60%, and the crystallization temperature resides between −50 °C and −70 °C.

## 1. Introduction

Neodymium-based rare earth polybutadiene rubber (Nd-BR) is characterized by a high cis-content, regular chain structure, and narrow molecular weight distribution. These attributes facilitate strain-induced crystallization during stretching, endowing the material with exceptional abrasion resistance, resilience, and mechanical properties. Such characteristics render Nd-BR suitable for the fabrication of high-performance, low rolling-resistance tires [1,2,3]. However, the ordered molecular structure that confers these advantageous properties also enhances the material’s crystallization ability at low temperatures, leading to deficiencies in low-temperature performance. For instance, Nd-BR may crystallize between −18 and −30 °C, resulting in increased permanent compressive deformation and reduced elasticity at low temperatures [4,5,6]. Therefore, investigating the crystallization processes and mechanisms of polybutadiene is critical for controlling its low-temperature crystallization and application.

To date, numerous scholars have conducted comprehensive assessments of the crystallization behavior and mechanisms of Nd-BR [7,8,9]. In accordance with crystallization kinetics principles, subjecting a sample to isothermal crystallization and establishing the relationship between the degree of crystallization and time via the Avrami equation provides insights into the crystallization kinetic parameters of Nd-BR [4,8,10,11]. Yang [4] studied the isothermal crystallization kinetics and crystalline morphology of Nd-BR by DSC and polarized optical microscopy (POM), revealing that the material crystallizes via three-dimensional spherulitic growth. The spherulite size increases with the *cis*-content, and the Avrami index *n* value during isothermal crystallization ranges between 2 and 3. Wrana [8] explored the relationship between crystallization rate and temperature in polybutadiene rubber via conventional differential scanning calorimetry (DSC) and fast differential scanning calorimetry (FDSC) and found that below −40 °C, the melting temperature of the initially formed crystals is relatively low, and the fraction of amorphous phase with restricted mobility (the rigid amorphous fraction) increased during crystallization at low temperatures, while the Avrami exponent is about unity and the maximum crystallization rate occurs at about −50 °C. The molecular structure of cis-content, branching degree, molecular weight, its distribution, and crystallization conditions can affect the crystallization of Nd-BR [12,13]. The branching degree is another factor affecting polymer crystallization; when the branching degree increases, the spherulitic growth rate decreases, and the spherulite size decreases. For instance, nickel-based polybutadiene rubber (Ni-BR) typically contains more short-chain branched structures, leading to decreased crystallization rates and degrees at −26 °C tested by DSC [5]. Zhang [14,15] studied the influence of a crosslinking network on crystallization by DSC; it was found that the crosslinking reaction restricts molecular mobility, thereby causing a noteworthy reduction in both crystallization temperature and enthalpy values. Saijo [16] delved into the crystallization behavior of crosslinked polybutadiene, investigating the impact of temperature and orientation on crystallization via small-angle X-ray scattering (SAXS) and wide-angle X-ray scattering (WAXS) and reported that weak to intermediate orientations indicate a decrease in long spacing during crystallization, which is accompanied by an almost constant lamellar thickness, as well as a significant time lag between the development of crystallinity, while The most pronounced feature of structural formation during crystallization under high orientations is an almost constant long period from nearly the beginning of the crystallization. The addition of fillers, exhibiting heterogeneous nucleation effects akin to those of nuclei, introduces crystallization nuclei, thereby resulting in a substantially higher crystallization temperature in the compounded rubber compared to rubber solely containing oil and lacking fillers [17,18]. Wunde [19] studied the effects of filler and other rubbers on the thermally induced crystallization of high-cis BR by dynamic mechanical analysis (DMA) and DSC and found that carbon black (CB) supports nucleation and growth of BR crystals and results in a pronounced increase in crystallization rate; on the other hand, in the blend of BR/styrene–butadiene rubber (SBR), crystallization is reduced for low SBR amounts and seems to disappear totally for 50/50 blend. Sun and Zhang [20] studied the effect of styrene–butadiene–styrene triblock copolymer (SBS) on non-isothermal crystallization kinetics and melting behavior of syndiotactic 1, 2-polybutadiene via DSC. Jeziorny, Ozawa, and Mo’s methods were used to analyze crystallization kinetics. The addition of SBS suppressed the crystallization behavior of s-PB with a decreased crystallization rate. In Nd-BR blends, the crystallization of BR may be accelerated or depressed. Yao [21] studied the phase structure and crystallization behavior of polyethylene in its blends with cis-1,4-butadiene rubber by DSC, scanning electron microscopy (SEM), and optical microscopy. It was found that the PE is finely dispersed in the BR matrix, and confined crystallization of PE occurred in small microdomains at relatively low temperatures. With the increase in domain size, the crystallization ability of PE increases while the confined crystallization decreases. Yang [22] examined the crystalline morphology of Nd-BR crystallized under isothermal conditions via SEM, discovering that within a range of −10 to −80 °C, polybutadiene rubber can exhibit six distinct crystalline morphologies, underscoring the effect of temperature on crystallization behavior. Cai [23] investigated the non-isothermal crystallization kinetics of trans-1,4-polybutadiene by DSC and determined using the Kissinger equation that the average crystallization activation energies for the hexagonal and monoclinic phases are −165.8 kJ/mol and −220.5 kJ/mol, respectively.

Other factors can also affect the crystallization of Nd-BR. Mitchell [24] studied the mechanical history effects on the crystallization of cis-l,4-polybutadiene dilatometric measurement of the volume contraction and revealed that mechanical forces can generate “quasi-indestructible” clusters in the polymer which survive well above the melting point and serve as especially active heterogeneous nuclei in the crystallization process. Makhiyanov [25] studied the glass transition and crystallinity of BR by DSC and reported that the glass-transition temperature of BR increases when cis-1,4 units is lower than 95% due to crystallization, and then it decreases with cis-1,4 units. Additionally, processed oil is a vital element affecting the crystallization behavior of rubber. Li [26] studied the crystallization of BR via linear dilatometer and reported the effect of oil amount on the processing and crystalline properties of Nd-BR, finding that an increase in oil content lengthens the semicrystalline time (*t*_1/2_) but does not significantly affect the Avrami index *n* value.

From the foregoing discussion, it is evident that the crystallization and application of BR rubber have garnered extensive attention, and a comprehensive understanding of its crystallization mechanisms and morphologies has been achieved. However, with advancements in catalytic systems and an increase in cis-content, the application of Nd-BR rubber is expanding, especially in low-temperature environments. This necessitates further investigation into the factors influencing its crystallization. The present study focuses on Nd-BR and explores the effects of small-molecule environmentally friendly treated distillate aromatic extract (TDAE) and high-molecular-weight polybutadiene–isoprene copolymer rubber (BIR) on the crystallization behavior of Nd-BR rubber. Here, both TDAE oil and BIR have very good compatibility with Nd-BR and cannot crystallize, so their effects on the crystallization of Nd-BR are dominantly the diluent effect. On the other hand, TDAE oil is usually used with BR in the tires. Therefore, both compounds are selected to study the influence of the dilution effect on Nd-BR crystallization. Here, the influence of both diluents on the crystallization behavior and kinetic parameters of Nd-BR blends are studied in order to reveal the effect of diluent on the crystallization kinetics of Nd-BR, which is of significance for the application of rubber in the low temperature and crystallization theory of rubber compounds.

## 2. Materials and Methods

### 2.1. Materials

Nd-BR with a grade of BR 9101N (density: 0.92 g/cm^3^; *M*_n_: 93,380 g/mol, molecular weight distribution (MWD): 3.719) was obtained from Dushanzi Petrochemical Co., Ltd. (Karamay, China); TDAE oil (density: 0.953 g/cm^3^; carbon type distribution for aromatic/naphthenic/paraffinic is 23%/39%/38%), was supplied by Hansheng Chemical Co., Ltd. (Ningbo, China); BIR (isoprene content: 18 wt.%; cis-structure%: 97%; *M*_n_: 126,468 g/mol and MWD: 2.72) was synthesized in-house.

### 2.2. Sample Preparation

Method for preparing Nd-BR/TDAE oil blends: A total of 200 g of Nd-BR rubber was plasticized on an open mill at a temperature of 50 °C. After the rubber was sufficiently masticated, TDAE oil was added incrementally, each time at a dosage of 10 PHR. Following thorough mixing, samples were retained. Oil–extended rubbers with oil contents of 10 parts per hundred of rubbers (PHR), 20 PHR, 30 PHR, 40 PHR, and 50 PHR were obtained and designated BR-10T, BR-20T, BR-30T, BR-40T, and BR-50T, respectively.

Method for preparing BR/BIR blends: BR and BIR were dissolved in hexane solvent according to their respective proportions. The homogeneous rubber solution was then poured into ethanol for coagulation. After air-drying for 12 h in a fume hood, the samples were dried in a vacuum oven at 60 °C and 1000 Pa for 24 h. Blended rubbers containing 100 wt.%, 80 wt.%, 60 wt.%, 40 wt.%, 20 wt.%, and 0 wt.% BR were labelled BR100, BR80, BR60, BR40, BR20, and BIR, respectively.

### 2.3. Testing and Characterization

The crystallization behavior was examined using a differential scanning calorimeter (DSC25, TA, New Castle, DE, USA) and analyzed by the TRIOS software v5.2.2.47561. Samples were cooled from 30 °C to −80 °C at rates of 5 or 10 °C/min, held for 1 min, and subsequently heated back to 30 °C at a rate of 10 °C/min.

Isothermal crystallization kinetics were investigated by rapidly cooling the samples to their crystallization temperature, holding them for 30 min, and then reheating them to 30 °C at a rate of 10 °C/min. Each sample was subjected to this procedure at multiple crystallization temperatures to observe the crystallization process at varying temperatures.

## 3. Results

### 3.1. Effect of Diluent Materials on Nd-BR Crystallization Behavior

**Figure 1:**
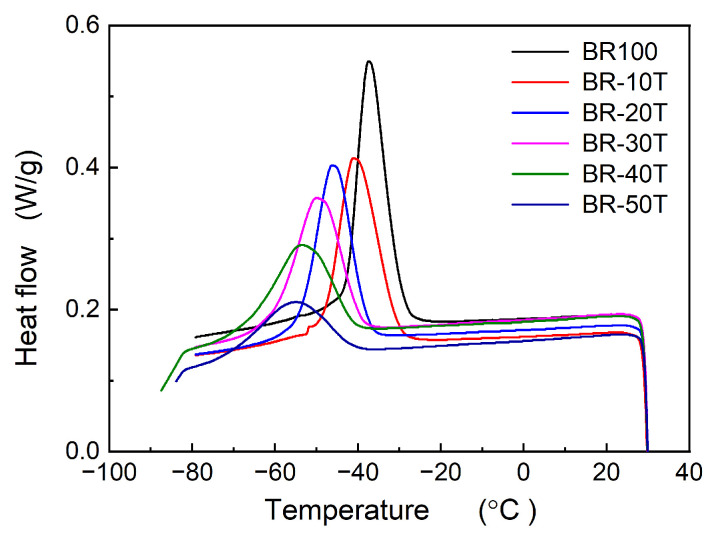
Crystallization procedure of Nd-BR/TDAE oil compounds; cooling rate: 5 °C/min.

**Figure 2:**
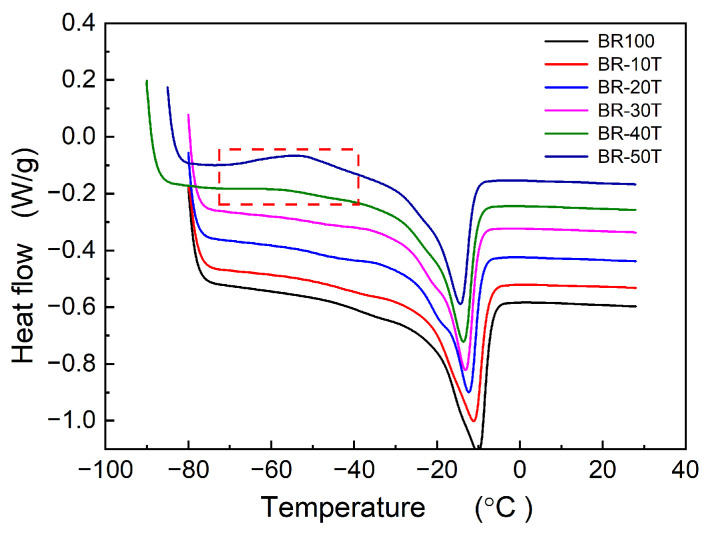
Melting procedure of Nd-BR/TDAE oil compounds; heating rate: 10 °C/min.

Figure 1 illustrates the crystallization behavior of Nd-BR rubber when compounded with TDAE oil. It was found that an increase in the content of TDAE oil led to a decrease in the crystallization temperature and degree of crystallinity of Nd-BR. As indicated by Table 1, within the experimental range, each 1 PHR addition of TDAE oil to Nd-BR caused the crystallization peak temperature (*T*_c,peak_) to decrease by approximately 0.37 °C. Additionally, for samples containing 40 PHRs of TDAE oil, the crystallization enthalpy (Δ*H*_c_) linearly decreased with increasing oil content; however, for samples containing 50 PHRs, Δ*H*_c_ of 16.79 J/g was markedly below the linear prediction of 23.74 J/g. Figure 2 provides the melting curves post-crystallization of Nd-BR, revealing that within the 0~50 PHR oil range, both the melting temperature (*T*_m_) and melting enthalpy (Δ*H*_m_) linearly decreased with increasing oil content (Table 1). Specifically, the *T*_m_ of BR-50T decreased by 4.25 °C compared to that of the native rubber. Furthermore, samples with 40 PHR and 50 PHR oil contents exhibited cold crystallization during the heating process, which formed an obvious exothermic peak of DSC, as highlighted in the red frame of Figure 2. with cold crystallization temperatures of −58.27 °C and −53.39 °C and cold crystallization enthalpies (Δ*H*_c,cold_) of 1.10 J/g and 4.78 J/g, respectively. It is evident that TDAE oil significantly inhibits the crystallization of Nd-BR, leading to concurrent reductions in *T*_c,peak_, crystallization rate, and Δ*H*_c_.

**Figure 3:**
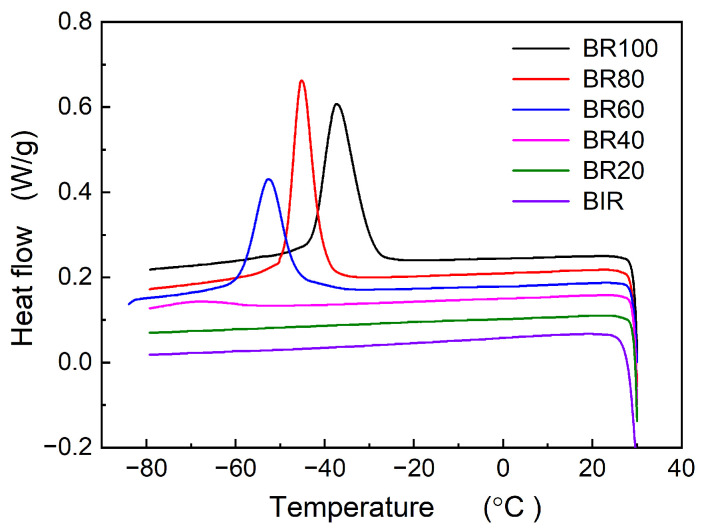
Crystallization procedure of Nd-BR/BIR rubber compounds; cooling rate: 5 °C/min.

**Figure 4:**
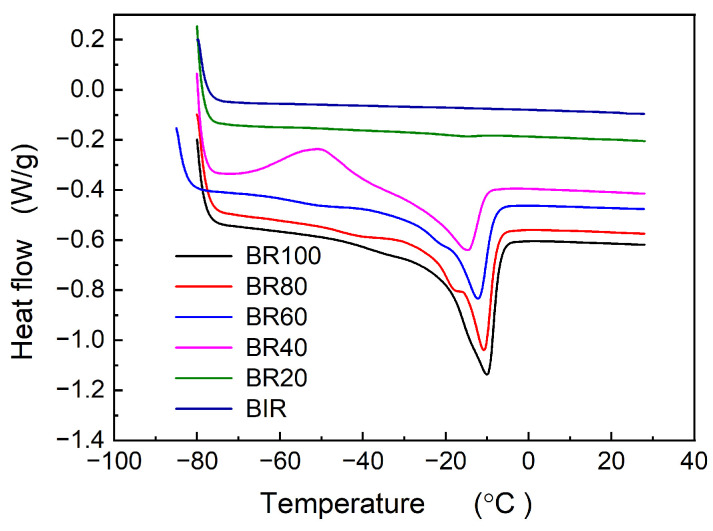
Melting procedure of Nd-BR/BIR rubber compounds; heating rate: 10 °C/min.

Figure 3 presents the crystallization behavior of the Nd-BR/BIR composite rubbers. It is evident that Nd-BR rubber exhibits pronounced low-temperature crystallization, whereas BIR rubber does not crystallize at low temperatures. When BIR rubber is blended with Nd-BR, the crystallization behavior of Nd-BR is significantly suppressed; no crystallization peaks appear for BR40 and BR20 during the cooling process. As indicated in Table 2, both *T*_c,peak_ and Δ*H*_c_ linearly increase with increasing Nd-BR content at 60 wt.%. Figure 4 shows the melting behavior of the Nd-BR/BIR composite rubbers, revealing the absence of a melting peak for BIR. Sample BR20 displays an extremely weak melting peak with a *T*_m, peak_ at −15.62 °C and Δ*H*_m_ merely at 0.551 J/g. Concurrently, both BR20 and BR40 exhibit cold crystallization, displayed as exothermic peaks before melting peaks in Figure 4. The cold crystallization temperature of BR40 and its enthalpy are −50.64 °C and 10.952 J/g, respectively. An investigation into the effect of cooling rates on cold crystallization shows that when the cooling rate changes from 5 °C/min to 10 °C/min, the *T*_m,peak_ for BR20 and BR40 increases, while Δ*H*_m_ decreases; both *T*_c,cold_ and Δ*H*_c,cold_ rise (Table 2). This is attributed to the accelerated cooling rate, which shortens the crystallization time at the same temperature and reduces the degree of crystallization, thereby intensifying the cold crystallization of BR during the heating process.

### 3.2. Effect of Diluents on Nd-BR Crystallization Kinetics

**Figure 5:**
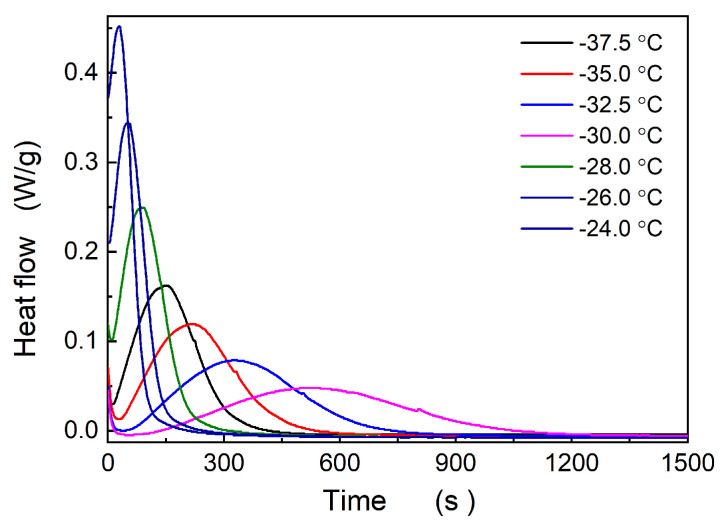
Crystallization procedure of Nd-BR at different temperatures.

Upon rapid cooling of the Nd-BR samples to the crystallization temperature, isothermal crystallization behavior can be obtained. Figure 5 illustrates the crystallization curves of pure Nd-BR at various temperatures. The relative degree of crystallization at different times can be calculated using the following equation:

(1) $$X_{\;c}=\frac{\int_{0}^{t}\frac{dH}{dt}dt}{\int_{0}^{\infty }\frac{dH}{dt}dt}$$

**Figure 6:**
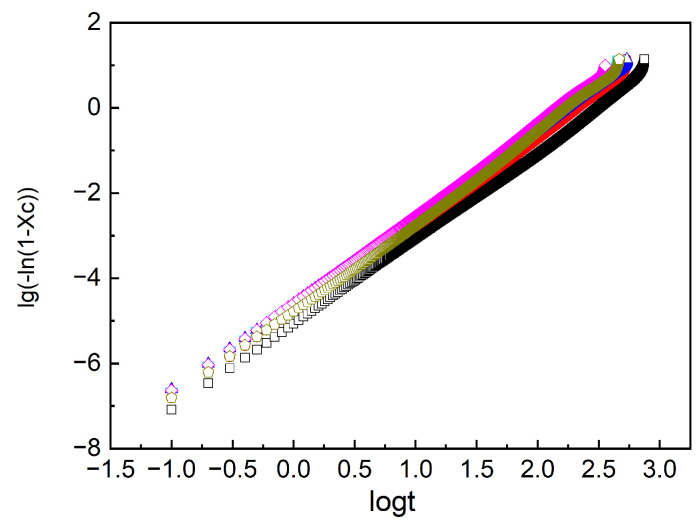
Evolution of the crystalline degree with time at different temperatures.

Here, *X*_c_ represents the crystallinity at time *t*, d*H*/d*t* is the heat flow rate, $\int_{0}^{t}\frac{dH}{dt}dt$ denotes the crystallinity at time *t*, and $\int_{0}^{\infty }\frac{dH}{dt}dt$ is the maximum crystallinity at the specified temperature. The relative crystallinity as a function of time, *X*_c_~*t*, is displayed in Figure 6, indicating that the crystallization rate decreases with increasing temperature. Based on the changes in the degree of crystallization over time, the crystallization kinetic parameters of the sample can be determined using the Avrami equation, given as

(2) $$1-X_{c}=exp(-kt^{n})$$

Here, *k* is the crystallization rate constant, and *n* is the Avrami exponent. Taking the logarithm of both sides of Equation (2) yields

(3) $$\ln\left[-\ln\left(1-X_{c}\right)\right]=ln\left(k\right)+n*ln(t)$$

**Figure 7:**
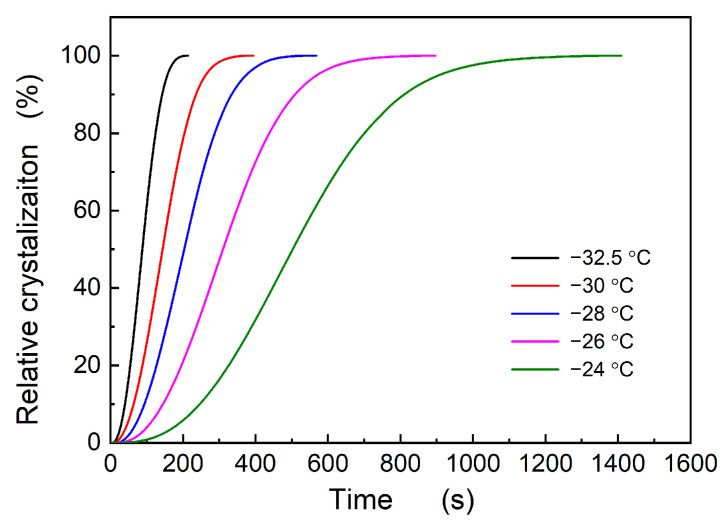
$\mathrm{lg}\left[-\ln\left(1-X_{c}\right)\right]$~lg(t) curves of the crystallization procedure.

The relationship between $\ln\left[-\ln\left(1-X_{c}\right)\right]$ and ln(t) yields the slope *n* and the intercept ln*k*, as illustrated in Figure 7. The relationship between *k* and *t*_1/2_ is as follows:

(4) t1/2=(ln2k)1/n

Isothermal crystallization of Nd-BR/TDAE oil composites at various temperatures yields crystallization kinetic parameters, as illustrated in Table 3. For the pure Nd-BR and BR-10T samples, *T*_m,peak_ increases with increasing crystallization temperature *T*_c_, whereas for BR-30T and BR-50T, *T*_m,peak_ decreases with increasing *T*_c_. At the same crystallization temperature, the *T*_m,peak_ for BR100 is approximately 0.57 °C higher than that for BR-10T, and the *T*_m,peak_ for BR-30T is 1 to 2 °C higher than that for BR-50T. This is attributed to the TDAE oil being dissolved between the rubber molecular chains, resulting in increased intermolecular distances and decreased crystallization temperatures. Additionally, the reduced molecular mobility at lower temperatures leads to decreased crystal sizes or increased crystal defects. According to Equation (4), *t*_1/2_ is related to the rate of crystallization. The *t*_1/2_ values obtained from Figure 6 and Equation (4) are presented in Table 3, with an error within 5%, indicating the reliability of the fitted data. The *t*_1/2_ for all samples increases as *T*_c_ increases, indicating a decline in the crystallization rate that aligns with the temperature dependence of the rate constant (*k*). The Avrami index *n* is related to the added TDAE oil; the *n* values for pure Nd-BR samples range from 2.56 to 2.68; and the addition of TDAE oil leads to a reduction in *n* values.

The *T*_m,peak_ of pure Nd-BR rubber is significantly influenced by the crystallization temperature *T*_c_, whereas the incorporation of BIR reduces the dependency of the melting point on *T*_c_, as indicated in Table 4. The crystallization kinetic parameters for various Nd-BR/BIR samples were obtained via Equation (3). The error between the original data and the *t*_1/2_ values calculated from Equation (4) is generally within 5%, corroborating the reliability of the fitted data. For BR80 and BR60, the *T*_c_ values are both above −50 °C, and *t*_1/2_ decreases as *T*_c_ increases. The crystallization temperature for BR40 is below −56 °C; *k* increases with an increase in crystallization temperature, and *t*_1/2_ decreases with an increase in *T*_c_, suggesting that higher temperatures facilitate a faster rate of crystallization.

## 4. Discussion

### 4.1. Effect of Dilution on Crystallization Kinetic Parameters of Nd-BR

The crystallization behavior of Nd-BR and its blends reveals that the addition of a non-crystalline second component alters the crystallization characteristics of Nd-BR. Specifically, the crystallinity, onset temperature, and peak crystallization rate all decrease with an increase in the second component. The process of polymer crystallization encompasses crystal nucleation and crystal growth, governed by the degree of undercooling Δ*T* (i.e., *T*_c_ − *T*_g_) and the capability of polymer chains to diffuse onto the crystal surface, respectively. For diluted Nd-BR samples, increased intermolecular spacing hinders nucleation, necessitating higher degrees of undercooling for crystal nucleation. In some cases, nucleation may not even occur within the limited cooling time, leading to cold crystallization behavior upon reheating, as depicted in Figure 2 and Figure 4.

For miscible polymers, the spherulite growth rate can be analyzed using the Lauritzen–Hoffman secondary nucleation theory [27] as follows:

(5) $$G=G_{0}\exp[\frac{-U^{*}}{R(T_{c}-T_{\infty })}]\exp[\frac{-K_{g}(T_{m}^{0}+T_{c})}{2T_{c}^{2}(T_{m}^{0}-T_{c})}]$$

Here, *K*_g_ is the nucleation parameter; U* is the activation energy for the transfer of large molecular chains to the crystal surface; *R* is the universal gas constant; $T_{m}^{0}$ is the equilibrium melting point; *T*_∞_ is the temperature at which molecules undergo freezing, typically expressed as (*T*_g_ − 30) °C; *T*_c_ is the crystallization temperature; and *G*_0_ is a constant. It follows that higher temperatures, approaching the polymer’s equilibrium melting point, result in a higher critical nucleation-free energy barrier and slower nucleation rates. Conversely, as the temperature nears the glass transition temperature, the activation energy barrier for molecular movement increases, thereby reducing the crystal growth rate. Thus, the overall crystallization rate exhibits a bell-shaped curve between *T*_g_ and *T*_m_, with the peak rate generally occurring near (*T*_m_ + *T*_g_)/2. For Nd-BR rubber, its *T*_g_ and *T*_m_ are −105 °C and −10.11 °C, respectively. Hence, the peak crystallization temperature *T*_c,max_ for Nd-BR is approximately −57.5 °C, consistent with literature reports [6].

According to Table 1, the onset crystallization temperature of Nd-BR/TDAE oil composites is generally higher than *T*_c,max_, and crystallization is primarily controlled by the degree of undercooling and nucleation density. The dilution effect of TDAE oil increases intermolecular spacing within the polymer, complicating nucleation and necessitating higher degrees of undercooling. As a result, the crystallization temperature decreases with increasing TDAE oil content; simultaneously, the half-peak width of the crystallization peak increases, indicating a reduced rate of crystallization with the addition of TDAE oil.

For Nd-BR/BIR rubber blends, Table 2 shows that when the Nd-BR content exceeds 50 wt.%, the *T*_c,onset_ variations are relatively minor. When the BIR content exceeds 50 wt.%, *T*_c,onset_ drops significantly, and crystallization may not even occur under the experimental conditions, as is the case with *T*_c,onset_ for BR40 being −55.86 °C. Isothermal crystallization experiments for BR40 were conducted in a temperature range of −70 to −56 °C. Therefore, the crystallization rate of BR40 is primarily governed by molecular mobility; higher temperatures within this range enhance crystallization capabilities, accelerating the rate as the temperature increases (Table 4). Table 3 and Table 4 indicate that the Avrami index *n* for different samples mainly ranges between 2 and 3. The *n* value correlates with the nucleation mechanism of crystallization. While BIR has a minimal effect on *n* for Nd-BR crystallization, even a small amount of TDAE oil causes a significant decrease in *n*, indicating a more substantial influence on the nucleation mechanism. Conversely, BIR exerts almost no effect on nucleation but influences the crystallization rate and temperature.

### 4.2. Effect of Dilution on the Activation Energy for Nd-BR Crystallization

Temperature plays a decisive role in the crystallization rate. According to the Arrhenius equation, one can correlate the crystallization rate with temperature. Combined with Equation (4), we have

(6) $$k=\frac{ln2}{t_{1/2}^{n}}=Aexp(\frac{-E_{a}}{RT})$$

Here, *A* is a constant, and *E_a_* is the crystallization activation energy. By manipulating Equation (6), one can obtain

(7) $$lnk=lnA+\frac{{-E}_{a,k}}{RT}$$

(8) $$\mathrm{or}\;lnt_{1/2}=\frac{ln(\frac{\ln2}{A})}{n}+\frac{E_{a,t(1/2)}}{nRT}$$

**Figure 8:**
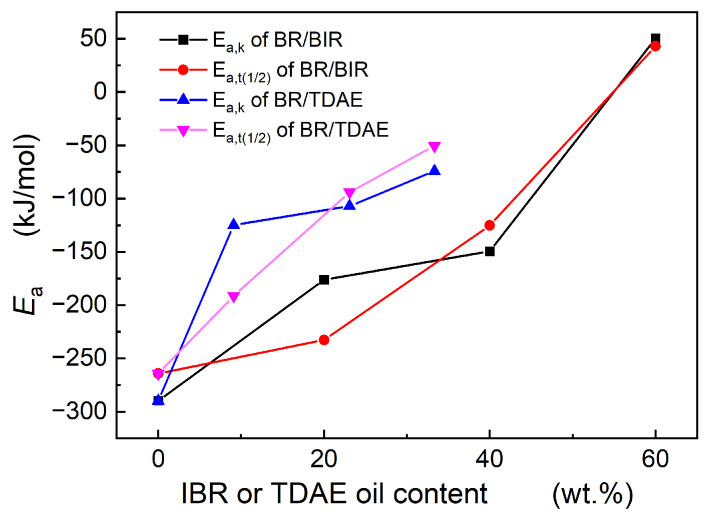
Activity energy of crystallization for BR/BIR and BR/TDAE oil compounds.

Consequently, the crystallization activation energy can be calculated via *k* or *t*_1/2_, denoted as *E_a_*_,k_ and *E_a_*_,t(1/2)_, respectively. Figure 8 presents the activation energy for Nd-BR composite systems. The activation energy data obtained from Equations (7) and (8) are relatively consistent; discrepancies likely arise from errors in curve fitting. The inclusion of TDAE oil or BIR leads to an increase in both the crystallization temperature and activation energy, thereby reducing the temperature sensitivity of the crystallization rate. At the same mass fraction, the activation energy of the Nd-BR/BIR composites is higher than that of the Nd-BR/TDAE oil composites, indicating a more substantial inhibitory effect of TDAE on the crystallization process.

This may be due to TDAE oil’s lower molecular weight and better compatibility with rubber molecules, leading to a stronger solvation effect. Although BIR exhibits excellent compatibility with Nd-BR, factors such as molecular weight make it challenging to disperse uniformly within Nd-BR molecular chains, resulting in microscopic concentration fluctuations. In Nd-BR/BIR composite rubber, when the BIR content reaches 60 wt.% (BR40), its crystallization activation energy is positive, in stark contrast to the negative activation energy at higher temperatures. As previously mentioned, the crystallization temperature for the BR40 sample is low, and isothermal crystallization experiments were conducted in a temperature range of −70 to −56 °C. Under these conditions, the crystallization process is primarily determined by molecular chain diffusion kinetics; increasing the temperature enhances molecular mobility, thereby elevating the crystallization rate. Consequently, the significant deviation in crystallization activation energy for BR40 compared to other samples is due to differing mechanisms governing crystal growth.

### 4.3. Influence of Dilution on Nucleation and Crystalline Growth in Nd-BR

**Figure 9:**
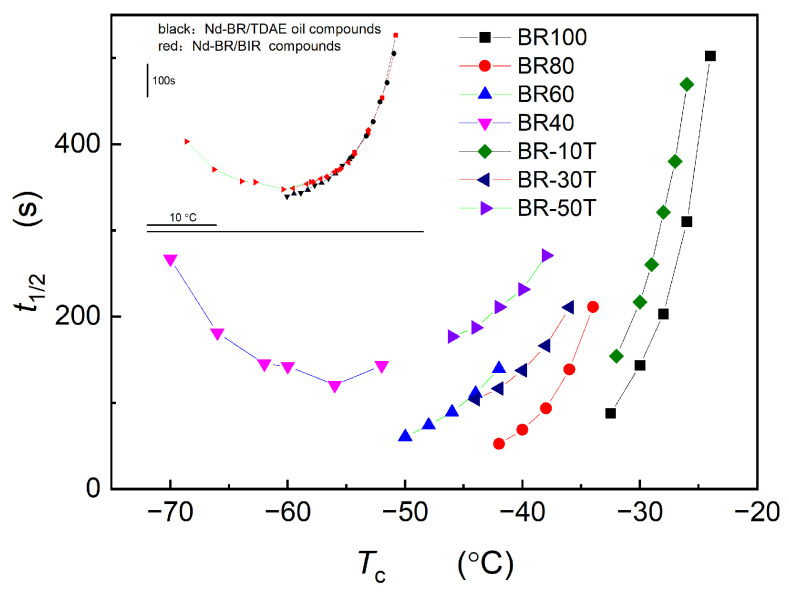
Dependence of *t*_1/2_ on *T*_c_ of Nd-BR and its compounds with BIR and TDAE oil.

Figure 9 delineates the relationship between *t*_1/2_ and *T*_c_ for various samples, revealing an increase in *t*_1/2_ as *T*_c_ rises above −50 °C, with a minimum value at approximately −55 °C. In the range of −70 °C to −55 °C, *t*_1/2_ decreases with an ascending *T*_c_. Examination of this relationship across different samples demonstrates consistent trends at varying temperatures, signifying a stable mechanism of Nd-BR crystallization. The primary variable is the relationship between the crystallization rate and temperature. As a result, overlaying the curves of *t*_1/2_ vs. *T*_c_ for different samples produces an aggregated curve. The inset in Figure 9 shows that at identical temperatures, the Nd-BR/TDAE oil composites manifest reduced *t*_1/2_ values. This can be attributed to the lower viscosity of TDAE oil, which facilitates the diffusion of Nd-BR molecular chains and accelerates the crystalline growth rate.

**Figure 10:**
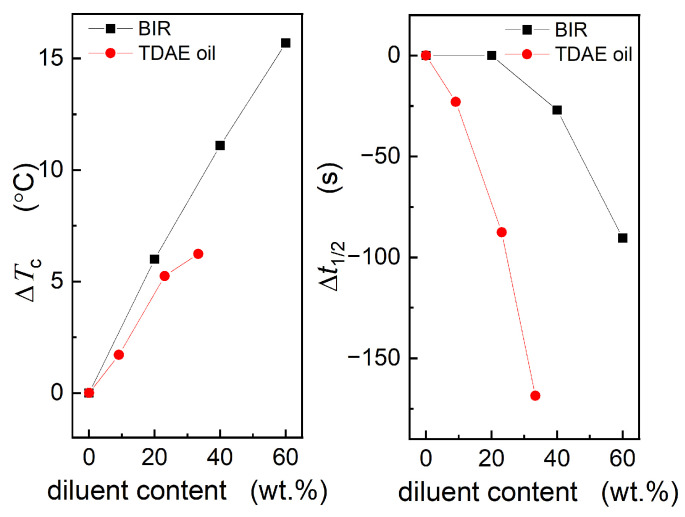
Dependence of Δ*T*_c_ and Δ*t*_1/2_ on the diluent content of BIR and TDAE oil.

The abscissa and ordinate in Figure 9 represent the crystallization temperature and crystallization rate, respectively. Therefore, the shifts in Δ*T*_c_ and Δ*t*_1/2_ indicate the effects of various diluents on nucleation and crystalline growth. As displayed in Figure 10, BIR exerts a more substantial effect on the nucleation temperature but a lesser effect on the crystallization rate than TDAE oil at identical concentrations. This observation is likely related to the differing viscosities and solvation capacities of the diluents. TDAE oil, possessing a lower viscosity than BIR, favors molecular diffusion and nucleation. However, its enhanced solvation capability hinders the insertion of Nd-BR molecular chains into the crystal lattice, thereby increasing the half-crystallization time and decelerating the crystallization rate.

## 5. Conclusions

The investigation into the effects of TDAE oil and BIR rubber on the crystallization behavior of Nd-BR rubber reveals salient results. Both TDAE oil and BIR rubber exhibit inhibitory effects on the nucleation of Nd-BR crystallization, leading to a decrease in crystallization temperature and melting point. Among the two, BIR exerts a more potent inhibitory effect on nucleation, whereas TDAE oil demonstrates stronger inhibition of the crystalline growth rate. However, the influence of both diluents on the crystallization kinetic parameters remains minimal, with their *n* values predominantly falling within the range of 2 to 3.

Nd-BR and its composites exhibit divergent mechanisms controlling crystalline growth under various crystallization conditions, and the effect of temperature on the crystallization rate varies accordingly. When the crystallization temperature is below −56 °C, Nd-BR possesses a strong nucleation capability, and the primary mechanism governing crystalline growth is molecular diffusion. Under these conditions, the rate constant for crystallization increases with increasing temperature, and the crystallization activation energy is positive. Conversely, at temperatures above −56 °C, Nd-BR exhibits enhanced motility; the crystalline growth is primarily nucleation-controlled. The rate constant for crystallization declines as the temperature increases, accompanied by a negative value for crystallization activation energy. Based on the above, it is concluded that both BIR and TDAE oil can depress the crystallization of Nd-BR and improve the deficiencies in the low-temperature performance of tires.

## Figures and Tables

**Table 1:** Crystalline parameters of Nd-BR/TDAE oil compounds.

	*T*_c,peak_ (°C)	*T*_c,onset_ (°C)	Δ*H*_c_ (J/g)	*T*_m,peak_ (°C)	Δ*H*_m_ (J/g)	*T*_c,cold_ (°C)	Δ*H*_c,cold_ (J/g)	*E*_a,k_ (kJ/mol)	*E*_a,t1/2_ (kJ/mol)
BR100	−37.44	−29.33	39.34	−10.11	43.75	/	/	−289.7	−264.3
BR-10T	−41.04	−30.42	35.52	−11.16	38.75	/	/	−124.8	−191.4
BR-20T	−46.28	−37.90	31.92	−12.35	34.36	/	/	/	/
BR-30T	−49.98	−39.58	29.04	−13.13	32.89	/	/	−107.1	−93.9
BR-40T	−53.61	−40.87	25.49	−13.68	30.75	−58.27	1.10	/	/
BR-50T	−55.23	−41.23	16.79 *	−14.36	27.17	−53.39	4.78	−74.2	−50.6

Note: cooling and heating rates of 5 and 10 °C/min, respectively; * much lower than the predicted value of 23.74 J/g.

**Table 2:** Crystalline results of BR/BIR compounds.

	*T*_c,peak_ (°C)	*T*_c,onset_ (°C)	Δ*H*_c_ (J/g)	*T*_m,peak_ (°C)	Δ*H*_m_ (J/g)	*T*_c,cold_ (°C)	Δ*H*_c,cold_ (J/g)	*E*_a,k_ (kJ/mol)	*E*_a,t1/2_ (kJ/mol)
BR100	−37.44	−29.33	39.34	−10.11	43.75	/	/	−289.7	−264.3
BR80	−45.22	−40.47	33.42	−10.80	34.46	/	/	−176.3	−232.8
BR60	−52.72	−46.35	29.20	−12.24	32.77	/	/	−149.5	−125.1
BR40	−68.67	−55.86	/	−14.72	20.37	−50.64	10.95	50.3	42.9
	/	/	/	−14.13 *	18.81 *	−48.28 *	16.33 *		
BR20	/	/	/	−15.62	0.55	−52.75	0.13	/	
				−14.66 *	0.32 *	−50.91 *	0.24 *		

Note: * cooling and heating rate: 10 °C/min; other data were obtained with cooling and heating rates of 5 and 10 °C/min, respectively.

**Table 3:** Parameters of crystallization kinetics for Nd-BR/TDAE oil compounds.

	*T*_c_ (°C)	*T*_m,peak_ (°C)	*t*_1/2_ (s)	(ln2k)1/n(s)	*n*	ln*k*
BR 100	−32.5	−10.44	87.70	86.22	2.62	−12.02
	−30	−10.67	143.50	142.98	2.56	−13.06
	−28	−10.22	203.10	207.98	2.66	−14.53
	−26	−9.64	310.10	319.04	2.57	−15.17
	−24	−8.97	502.30	520.03	2.68	−17.11
BR-10T	−32	−11.64	154.14	149.52	2.30	−11.90
	−30	−11.26	216.72	208.57	2.16	−11.90
	−29	−11.05	260.40	250.26	2.14	−12.18
	−28	−10.79	321.12	316.32	2.09	−12.41
	−27	−10.47	380.10	358.26	1.89	−11.47
	−26	−10.18	469.44	444.50	1.90	−11.97
BR-30T	−44	−13.09	104.22	106.23	2.48	−11.93
	−42	−13.19	116.58	117.54	2.32	−11.42
	−40	−13.36	137.70	140.12	2.36	−12.02
	−38	−13.72	166.32	167.18	2.30	−12.13
	−36	−14.63	210.78	213.73	2.35	−12.96
BR-50T	−46	−14.11	176.82	176.48	2.13	−11.37
	−44	−14.14	187.20	186.43	2.13	−11.49
	−42	−14.37	210.90	211.18	2.12	−11.70
	−40	−14.83	231.60	231.77	2.15	−12.07
	−38	−15.69	270.96	273.94	2.16	−12.48

**Table 4:** Parameters of crystallization kinetics for Nd-BR/BIR rubber compounds.

	*T*_c_ (°C)	*T*_m,peak_ (°C)	*t*_1/2_ (s)	(ln2k)1/n(s)	*n*	ln*k*
BR80	−42	−10.19	52.30	53.09	3.07	−12.55
	−40	−10.19	68.80	73.54	2.95	−13.06
	−38	−10.37	93.60	100.82	2.76	−13.10
	−36	−10.73	138.70	143.25	2.90	−14.76
	−34	−11.68	211.30	211.62	2.83	−15.54
BR 60	−50	−11.59	60.60	60.17	2.73	−11.54
	−48	−11.51	74.00	75.51	2.81	−12.50
	−46	−11.45	89.30	94.29	2.86	−13.35
	−44	−11.41	110.70	116.18	2.90	−14.16
	−42	−11.5	139.60	141.77	2.79	−14.18
BR 40	−70	−14.76	267.10	266.43	2.09	−12.02
	−66	−14.63	181.50	180.02	2.12	−11.37
	−62	−14.5	145.30	146.57	2.11	−10.89
	−60	−14.33	142.50	141.73	2.13	−10.94
	−56	−14.17	120.60	122.98	2.18	−10.87

## Data Availability

The data that support the findings of this work are available from the corresponding author upon reasonable request.
